# Investigating the role of disulfidptosis related genes in radiotherapy resistance of lung adenocarcinoma

**DOI:** 10.3389/fmed.2024.1473080

**Published:** 2024-10-23

**Authors:** Xiaoxia Pan, Hongyan Qian, Zhouna Sun, Qiong Yi, Ying Liu, Gangzhi Lan, Jia Chen, Gaoren Wang

**Affiliations:** ^1^Cancer Research Center Nantong, Affiliated Tumor Hospital of Nantong University and Medical School of Nantong University, Nantong, China; ^2^Department of Radiation Oncology, Nantong Tumor Hospital, Affiliated Tumor Hospital of Nantong University, Nantong, China; ^3^Department of Oncology Internal Medicine, Nantong Tumor Hospital, Affiliated Tumor Hospital of Nantong University, Nantong, China

**Keywords:** LUAD, radiotherapy, disulfidptosis, prognosis, tumor immune microenvironment

## Abstract

**Background:**

Radiotherapy resistance is an important reason for high mortality in lung cancer patients, but the mechanism is still unclear. Dysregulation of cell proliferation and death plays a crucial role in the onset and progression of lung adenocarcinoma (LUAD). In recent times, a novel form of cellular demise called disulfidptosis, has attracted increasing attention. However, it is unclear whether the radiation-related disulfidptosis genes have prognostic role in LUAD.

**Methods:**

A complete suite of bioinformatics tools was used to analyze the expression and prognostic significance of radiation-related disulfidptosis genes. Afterward, we investigated the predictive significance of the risk signature in tumor microenvironments (TME), somatic mutations, and immunotherapies. In addition, we conducted a series of experiments to verify the expression of differentially expressed radiotherapy related disulfidptosis genes (DERRDGs) *in vitro*.

**Results:**

A total of 88 DERRDGs were found. We constructed and validated a novel prognostic model based on PRELP, FGFBP1, CIITA and COL5A1. The enrichment analysis showed the DERRDG affected tumor prognosis by influencing tumor microenvironments (TME) and immunotherapy. And we constructed nomogram to promote clinical application. In addition, q-PCR confirmed the significant differences in the expression of prognostic genes between A549 irradiation-resistance cell and A549. Finally, western-blot, IHC staining, and small interference experiment suggested that PRELP may be a potential biomarker for radiotherapy resistance, whose low expression was associated with poor outcomes in LUAD patients.

**Conclusion:**

This study reveals the signature and possible underlying mechanisms of DERRDGs in LUAD and discovered the key gene PRELP, which helps to identify new prognostic biomarkers and provides a basis for future research.

## 1 Introduction

According to the 2024 report from the World Health Organization, lung cancer is the primary reason for the incidence and mortality of cancer, making up 12.4% of global cancers and 18.7% of cancer-related fatalities (1). Among them, lung adenocarcinoma (LUAD) is the most common subtype of lung cancer worldwide (2, 3). Radiotherapy is one of the most important non-surgical treatments for LUAD, but the effectiveness varies. Decreased sensitivity to radiotherapy is the main reason for treatment failure in LUAD patients, leading to tumor recurrence or distant metastasis in more than 40% of patients (4, 5). Therefore, it is a crucial task to study the mechanism of decreased radiosensitivity and find key target genes to improve radiotherapy for LUAD patients.

Multiple forms of cell death were implicated in the modulation of radiotherapy sensitivity, including apoptosis (6), ferroptosis (7), autophagy (8), etc. Recently, a novel form of cell death-disulfidptosis has been reported, which is not mitigated by other cell death inhibitors, nor is it attributed to intracellular ATP depletion, but is enhanced by thiol oxidants such as dimethylamine. During glucose deprivation, cells with high expression of cystine transporter solute carrier family member 11 (SLC7A11), reduced nicotinamide adenine dinucleotide phosphate (NADPH), leading to an atypical buildup of cystine and other disulphide linkages, thereby promoting aberrant disulfide bond formation within the actin cytoskeleton. This eventually leaded to actin network breakdown and disulfide bond toxicity, resulting in disulfidptosis (9, 10). Furthermore, it has been demonstrated that disulfide metabolism has the capacity to influence the immune microenvironment (11). Multiple investigations have verified the influence of disulfidptosis-related genes on the prognosis on patients with LUAD (12–15), however, their role and underlying mechanism in radiotherapy sensitivity is still unknown.

In this research, we identified four differentially expressed radiotherapy related disulfidptosis genes (DERRDGs) in LUAD. We constructed a prognostic prediction risk model and simultaneously analyzed the causes of prognostic differences by immunoinfiltration analysis and immunotherapy analysis. After performing a sequence of studies and verifications, we assessed the prediction efficacy of the model. Ultimately, we pinpointed PRELP as a key gene that impacted the efficacy of radiotherapy in LUAD. As a result, we believed that disulfidptosis patterns could serve as a potential biological target for radiotherapy in LUAD, and the results of our research are anticipated to provide valuable diagnostic and treatment approaches for people with LUAD.

## 2 Methods and materials

### 2.1 Data collection and sample processing

RNA sequencing, survival data, and clinical data were collected from the Cancer Genome Atlas (TCGA) database hosted on the Xena platform at the University of California Santa Cruz (UCSC) (16).^1^ After data cleaning and standardization, a total of 500 tumor tissues and 59 para-carcinoma tissue were included. 19 complete response (CR) or partial response (PR) cases after radiotherapy were defined as radiosensitivity (RS) patients and 31 stable disease (SD) or progressive disease (PD) cases after radiotherapy were defined as radioresistance (RR) patients. The radiotherapy dataset GSE162945 was downloaded from the GEO database to exclude squamous cell carcinoma and incomplete information. A total of 16 LUAD patients (8 RR and 8 RS) were identified. In addition, GSE50081 (127 patients with LUAD) and GSE72094 (397 patients with LUAD) were used as external validation data set (17).^2^ Liu’s study obtained 24 disulfidptosis-related genes (FLNA, FLNB, MYH9, TLN1, ACTB, MYL6, MYH10, CAPZB, DSTN, IQGAP1, ACTN4, PDLIM1, CD2AP, INF2, SLC7A11, SLC3A2, RPN1, NCKAP) (9). Our study examined the association among 24 genes in TCGA-LUAD.

### 2.2 Identification of DERRDGs involved in LUAD

Based on the expression of disulfidptosis-related genes in each sample, We employed the Consensus ClusterPlus package of R software to categorize 500 LUAD samples into discrete molecular clusters (18). The K-Means algorithm was employed for 1,000 times of consistent clustering (Euclidean distance), with a resampling rate of 80%. The optimal number of clusters was determined using the experience accumulation distribution function graph. Meanwhile, the ggplot2 package of R software was used for principal component analysis (PCA) (19). Radiation-related genes (DRGs) identify was performed on the GSE162945 dataset using the R package “limma” to analysis genes that were between RR and RS. Simultaneously, the disulfidptosis-related differentially expressed genes (DEGs) in TCGA-LUAD between two clusters of disulfidptosis were identified. The criterion of differential expression was established as | log2FC| > 1 and *P* < 0.05. The resulting gene sets were compared to identify 88 differentially expressed radiotherapy related disulfidptosis genes (DERRDGs) through intersection analysis.

### 2.3 Construction and verification of RiskScore model

We built a risk model using 500 LUAD samples with survival information from the TCGA-LUAD cohort. First, through univariate Cox regression, we have pinpointed OS-associated genes in the TCGA-LUAD cohort. Following this, the least absolute shrinkage and selection Operator (LASSO) algorithm was applied to OS related genes within the TCGA-LUAD queue (20). Finally, the signature of disulfidptosis was constructed by stepwise Cox regression algorithm. The risk score was calculated based on the following formula:


$$R\,i\,s\,k\,S\,c\,o\,r\,e:\sum_{i=1}^{n}=C\,o\,e\,f\,\left(g\,e\,n\,e\right)*E\,x\,p\,r\,e\,s\,s\,i\,o\,n$$

Patients exceeding the median risk score were designated as high-risk subgroup, whereas others were categorized into low-risk subgroup. Two external validation sets GSE50081 and GSE72094 were applied to test and assess the prevalence of risk characteristics. 1, 3, and 5 year receiver operating characteristic (ROC) curves were plotted using R software’s Time ROC package, and the corresponding area under the curve (AUC) over time was computed as a measure of the predictive accuracy of the model. In order to thoroughly evaluate the disparity in prognosis between subgroups categorized as low and high-risk, we performed Kaplan–Meier (KM) survival analyses using the R software survival kit (*P* < 0.05).

### 2.4 GO, KEGG, GSEA and GSVA analysis

The aim is to delve into the potential mechanisms and pathways between RS and RR, the R software packages “clusterProfiler,” “enrichment plot,” “org.hss.egg.db,” “limma” and “ggplot2” were used for Gene Ontology (GO), Kyoto Encyclopedia of Genes and Genomes (KEGG), gene-set enrichment analysis (GSEA) and gene-set variation analysis (GSVA) analysis.

### 2.5 Protein-protein interaction (PPI) analysis

Utilizing the STRING database,^3^ we delved into potential disulfidptosis-related genes interactions, employing a threshold of a minimum interaction score of 0.15. Subsequently, PPI networks were crafted and visualized via Cytoscape (version 3.9.1).

### 2.6 Analysis of immune and somatic variation

The immune infiltration algorithm primarily utilized the ssGSEA algorithm from the R-Package GSVA (21). It employed markers from 24 immune cells, as supplied in the immunity article (22), to compute the immunological infiltration. The TIDE algorithm was employed to forecast potential immunotherapy responses, offering insights into treatment efficacy. From TISDE database,^4^ a dataset of 28 kinds of immune infiltrating cells and their associated 782 genes was obtained, and the enrichment of 28 immune infiltrating cells in tumor samples was evaluated by single sample gene set enrichment analysis (ssGSEA). The abundance of six types of immune infiltrating cells (B cells, macrophages, DC, neutrophils, CD4 T cells and CD8 T cells) was calculated using TIMER immune infiltration analysis. In addition, esteemed algorithms like ESTIMATE, CIBERSORT, and MCPCounter were employed to validate the practicality of ssGSEA and TIMER, ensuring their reliability and efficacy. Finally, we also analyzed the differential expression of 9 immune checkpoint related genes in the high and low groups (23).

We utilized waterfall maps to precisely identify and compared genes exhibiting a higher somatic mutation frequency in both high-risk and low-risk groups.

### 2.7 Construction and evaluation of the nomogram

To enhance the application of DERRDG to forecast the prognosis of LUAD, we leveraged the “rms” R package to formulate a nomogram model. The “rms” program was utilized to generate calibration curves that demonstrate the concordance between projected 1, 3, and 5 year end events and observed outcomes. Then, we mapped out calibration curves, ROC curves, and decision curve analysis (DCA) graphs to gain further insights. Moreover, we also used RiskScore as a separate variable in order to do univariate and multifactorial independent prognostic analyses, along with other clinically significant prognostic aspects.

### 2.8 Comprehensive bioinformatics analysis of key gene PRELP

Initially, we examined the correlation among the four DERRDGs, then analyzed the similarity between them, and elucidated the alterations in the expression of the remaining three model genes as changes in PRELP expression. R software established a binary Logistic model to analyze the relationship between DERRDGs gene expression level and clinical features between RS and RR patients in the TCGA-LUAD-radiotherapy cohort, presented by grouping violin diagram. KM-plot platform^5^ analyzed prognosis significance.

### 2.9 Weighted correlation network analysis (WGCNA) and single cell sequencing analysis

WGCNA was performed according to PRELP gene expression level. Then, we embarked on the determination of an optimal soft threshold for the data to ensure that the gene interactions conform to the scale-free distribution to the greatest extent possible. The proximity and resemblance of genes were computed, and the clustering tree was constructed. The dynamic tree cutting algorithm is segmented the modules and combined the similar modules. We assessed the Pearson correlation between each sample traits and module, and picked the module genes with the highest absolute value for further research.

We performed a rigorous screening process on single-cell RNA sequencing data from GSE153935 dataset on the TISCH platform^6^ to identify and extract key information fragments.

### 2.10 Colony formation assay

A549 cells and A549 irradiation-resistance cell (A549IR) were inoculated into a six-well plate at a density of 150, 400, 800, or 1,500 cells/Wells and exposed to 0, 2, 4, or 6 Gy, respectively. After about 12–14 days, the cells were washed with PBS, fixed with 4% paraformaldehyde, and finally counted with crystal violet. The survival curve is generated according to the linear quadratic model.

### 2.11 Quantitative polymerase chain reaction (q-PCR)

A549IR, A549, H1299, H1975, PC9 cell cultures and 8 LUAD cancer tissues were collected for qPCR detection were collected for q-PCR. The extraction of total RNA was performed using TRIzol reagent (Thermo Fisher SCIENTIFIC, USA). The EvoM-MLV reverse transcription kit (Accurate Biology, China) was utilized for the mRNA reverse transcription process (24). The primers used in this study were purchased from Sangon Biotech, and the primers sequence is shown in Table 1.

**TABLE 1:** The primer sequence of 4 genes.

Gene	Primer	Sequence (5′–3′)
PRELP	Forward	TTAACCTGGACAACAACCGAAT
	Reverse	CTGGTTCTTCTCCATGTAGAGG
FGFBP1	Forward	CCAGGAAGGAGAAAACAGAGAT
	Reverse	TCTTCCTCTGGTTTGCCATATC
CIITA	Forward	TTGGGCAGAAAAGTCAGAAAAG
	Reverse	CTCAACGAGGAACTGGAGAAAG
COL5A1	Forward	CGTATGATGACCTCACCTATGG
	Reverse	CGTAGTAGTTCTCGTCAAGGTT

### 2.12 Western blot (WB)

The cells were washed twice with PBS and lysed with phenylmethylsulfonyl fluoride (1:100, Beyotime, Shanghai, China) combined with cell lysis buffer. The proteins were then isolated by electrophoresis and transferred to a polyvinylidene fluoride (PVDF) membrane (Invitrogen, USA). Following a 2-h blocking step with 5% skim milk, incubation with PRELP antibody (1:1,000, Proteintech, China) was conducted overnight at 4°C. The membrane was washed and incubated with the corresponding secondary antibody (1:1,000, Proteintech, China). Finally, the enhanced chemiluminescence method was used for visualization, and the relative expression level of the protein was normalized with GAPDH as the internal control.

### 2.13 Immunohistochemistry (IHC)

Tumor tissues were obtained from the Hospital Affiliated with Nantong University. Sections were heated at 95°C in 0.01 M citric acid buffer (pH = 6.0). The tissue sections were incubated with primary anti-PRELP antibody (dilution 1:100; Cat No: 23783-1-AP, Proteintech, China), followed by treatment with an appropriate detection system. High-resolution images of stained sections are captured using a scanning microscope (Nikon, Japan). The evaluation of PRELP staining was performed by two independent pathologists who were not privy to the corresponding clinical information.

### 2.14 Immunofluorescence assay

The cells were plated in polylysine coated glass, incubated for 24 h, and then exposed to a 2 GY X-ray line. After incubation for 6 h, the cells were stained with DNA damage detection kit (product number: C2038S, Beyotime, Shanghai, China). Nikon microscopy was used to detect and quantify the γ-H2AX signal to determine whether DNA was damaged in the nucleus. At least 50 nuclei were evaluated in each group.

### 2.15 Statistical analysis

Data processing, statistical analysis, and charting were performed using the R4.1.0 program. When both groups exhibited a normal distribution and had a defined mean square error, the *t*-test was employed. When both groups were non-normal distribution, Wilcoxon rank sum test was used. The Kaplan–Meier method was employed for a thorough analysis of the prognosis and the resulting survival curve. Differences between groups were analyzed by logrank test or Cox regression analysis. The ROC curve was employed as a metric to assess the efficacy of the model’s predictive capabilities. *P* < 0.05 was statistically significant (ns: *P* > 0.05, *P < 0.05, **P < 0.01, *P < 0.001, *P < 0.0001).

## 3 Results

### 3.1 Screening and functional enrichment analysis of DRGs

**FIGURE 1:**
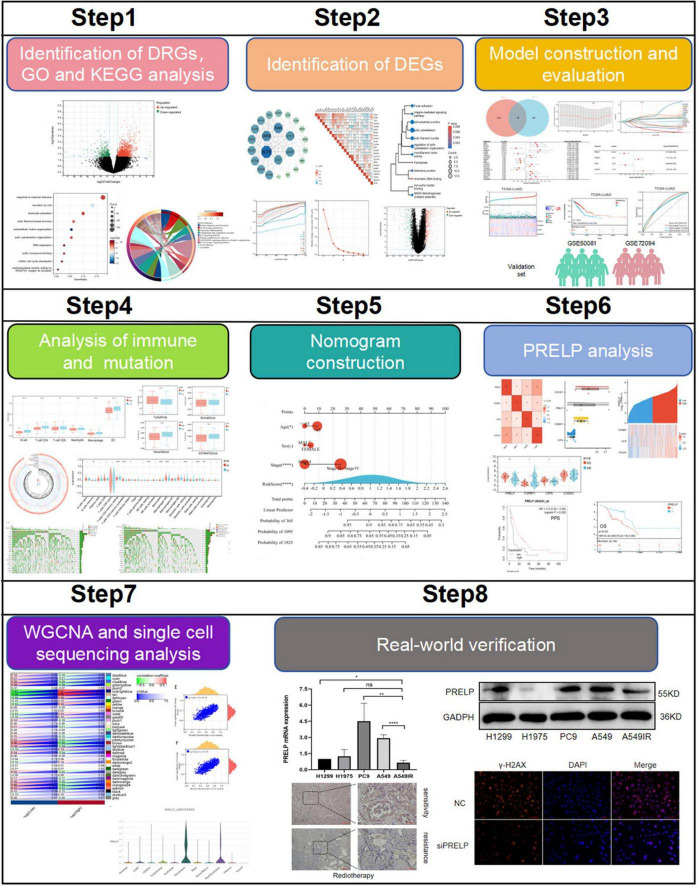
Flow chart of this research. (ns: *P* > 0.05, *P < 0.05, **P < 0.01, ***P < 0.001, ****P < 0.0001).

**FIGURE 2:**
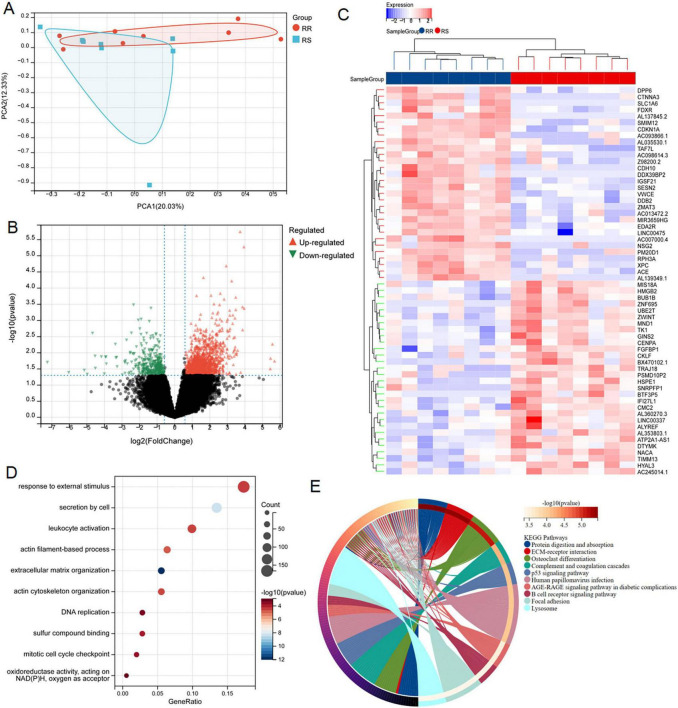
Screening and functional enrichment analysis of DRGs between RR and RS. **(A)** PCA analysis of GSE162945 database. **(B,C)** Volcano and heat maps of differentially expressed genes between RR and RS in the GSE162945 database. **(D,E)** GO and KEGG enrichment analysis of differentially expressed genes.

The flowchart of the study was shown in Figure 1. In order to identify the genes associated with RR, PCA demonstrated that samples from the two groups (8 RR and 8 RS samples in GSE169245) were significantly separated from each other and clustered well (Figure 2A), limma analysis identified 1365 up-regulated DRGs and 396 down-regulated DRGs, and the heat map showed the TOP30 up-regulated DRGs and down-regulated DRGs, respectively (Figures 2B, C). Next, GO analysis enriched the leukocyte activation, actin cytoskeleton organization, DNA replication, sulfur compound binding, mitotic cell cycle checkpoint and oxidoreductase activity, acting on NADPH, oxygen as acceptor (Figure 2D). KEGG was enriched into p53 signal pathway, B cell receptor signaling pathway, focal adhesion etc (Figure 2E). These results hinted disulfidptosis may be the important biological process in the radiotherapy sensitivity of patients with LUAD.

### 3.2 Consensus cluster analysis and identification of DERRAGs

**FIGURE 3:**
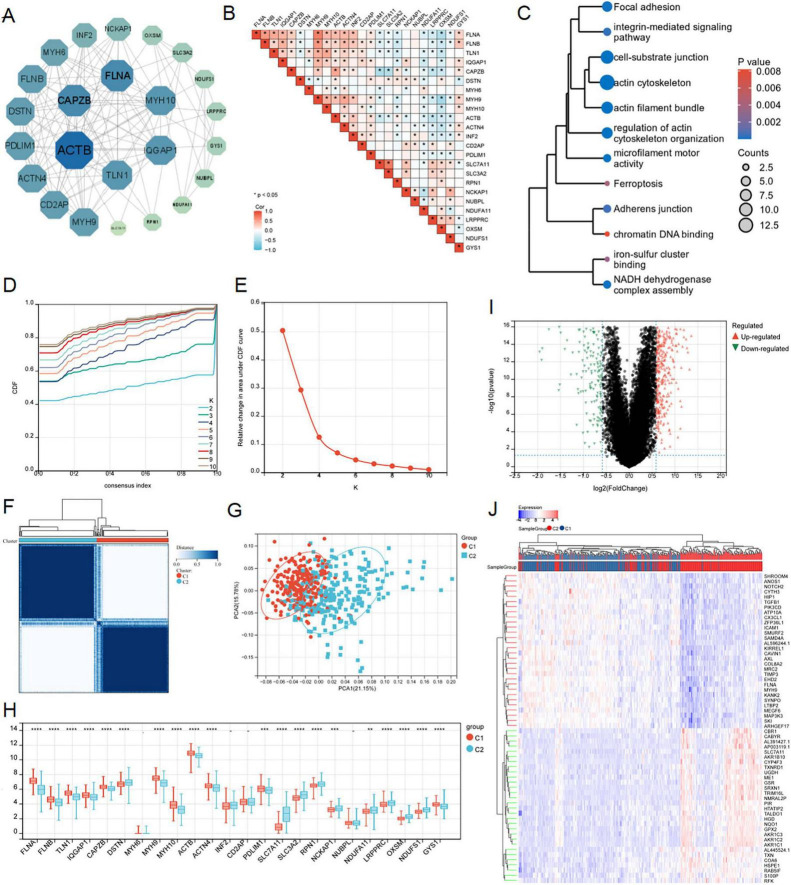
Consensus clustering analysis and identification of disulfidptosis-related genes in LUAD. **(A)** PPI interactive network of disulfidptosis-related genes using string database. **(B)** Heat map showed related genes interactions in LUAD. **(C)** GO and KEGG enrichment analysis of related genes. **(D–F)** The average consistency within the group was the highest to determine the optimal cluster number (*K* = 2) for classification. **(G)** PCA was performed on 500 LUAD patients, with each point representing a sample. **(H)** Differential expression analysis of disulfidptosis-related genes among different subgroups in the TCGA-LUAD cohort. **(I,J)** Volcanic maps and heat maps showed limma analysis between different subtypes (ns: *P* > 0.05, *P < 0.05, **P < 0.01, ***P < 0.001, ****P < 0.0001).

We successfully retrieved 24 disulfidptosis-related genes from Liu’s study, which were depicted in the PPI network (Figure 3A). Subsequently, we performed a correlation analysis based on the expression levels of disulfidptosis-related genes, and the results showed that the expression level of these genes in LUAD was almost correlated negatively (Figure 3B). In addition, GO and KEGG pathway enrichment analysis were employed for them. As shown in Figure 3C, these genes were involved in regulation of actin cytoskeleton organization, NADH dehydrogenase complex assembly, iron-sulfur cluster binding, focal adhesion, chromatin DNA binding and so on. This result reminded again the importance of disulfidptosis in radiosensitivity.

According to the expression patterns of 24 disulfidptosis-related genes, the samples in the TCGA-LUAD dataset were coherentially clustered to identify the sample groups with similar expression patterns. When the number of clusters is *K* = 2, the average consistency within the group was the highest. Thus, the sample was segmented into two subtypes (Figures 3D–F). The distribution pattern of PCA showed that the sample could be completely distinguished into clusters 1 and 2 (Figure 3G). We analyzed expression levels of disulfidptosis-related genes in the two clusters, finding significant variations in 20 genes (Figure 3H). The limma package successfully identified DEGs between the C1 and C2 subtypes, comprising 453 up-regulated and 193 down-regulated genes (Figures 3I, J).

### 3.3 Construction and validation of DERRDGs risk model

**FIGURE 4:**
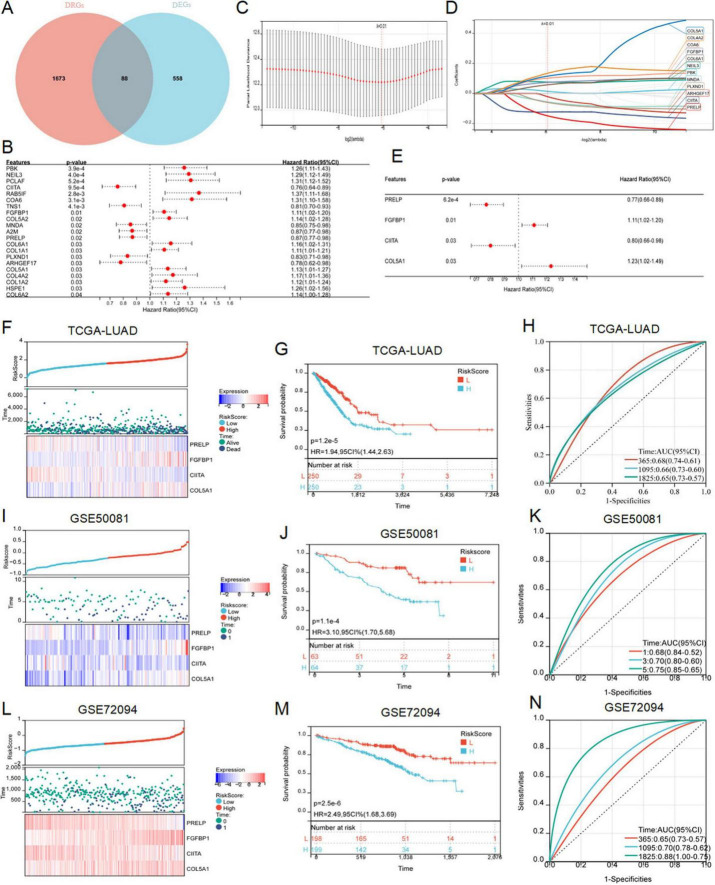
Construction and verification of RiskScore model. **(A)** The Venn diagram showed the intersection genes of DRGs and DEGs. **(B)** Forest maps showed the results of univariate Cox regression analysis. **(C)** Trajectories of variables for Lasso regression analysis. **(D)** Selection of variable coefficients for Lasso regression analysis. **(E)** Multivariate cox regression analysis of forest maps. RiskScores, survival time, survival status, and gene expression of TCGA-LUAD **(F)**, external set GSE50081 **(I)**, and external set GSE72094 **(L)**. Kaplan–Meier analysis showed the prognostic significance of the risk model in TCGA-LUAD **(G)**, GSE50081 **(J)**, and GSE72094 **(M)**. ROC curves were used to assess the accuracy of the model in predicting prognosis in TCGA-LUAD **(H)**, GSE50081 **(K)**, and GSE72094 **(N)**.

To further explore the relationship between disulfidptosis and radiotherapy response in LUAD patients, we crossed DRGs and DEGs and identified 88 common DERRDGs (Figure 4A). 88 DERRDGs were analyzed by univariate cox method to evaluate the prognostic significance and obtained 21 OS-related genes (Figure 4B). Then, the 21 genes were subjected to lasso-cox regression analysis (Figures 4C, D), and followed by multivariate cox regression analysis (Figure 4E). Under the optimal regularization parameters, 4 genes (PRELP, FGFBP1, CIITA, COL5A1) were finally screened. The prediction model is calculated as follows:


RiskScore=-0.213300882418068*PRELP+0.09055911612

⁢4251*FGFBP1-0.122074606361669*CIITA+

⁢0.158512593395885*COL5A1

LUAD samples in the TCGA cohort was divided into high-risk and low-risk subgroups according to the median to facilitate the following study. Survival analysis showed that patients in low-risk subgroup had a significantly better prognosis than those in high-risk subgroup (Figures 4F, G). In addition, the results of the risk curves and survival state graphs of two external validation sets, GSE50081 and GSE72094, showed that the survival outcome of the low-risk group was better than that of the high-risk group (Figures 4I, J, L, M). The AUC values of 1, 3 and 5 year ROC curves in LUAD were 0.68, 0.66 and 0.65, respectively (Figure 4H), 0.68, 0.70, and 0.75 in the GSE50081 validation set (Figure 4K), and 0.65, 0.70, and 0.88 in the external validation set GSE72094 (Figure 4N). These findings suggested that the prognostic model of DERRDGs riskscore was highly accurate in predicting outcomes in both groups of patients.

### 3.4 Potential mechanism of DERRDGs in LUAD

**FIGURE 5:**
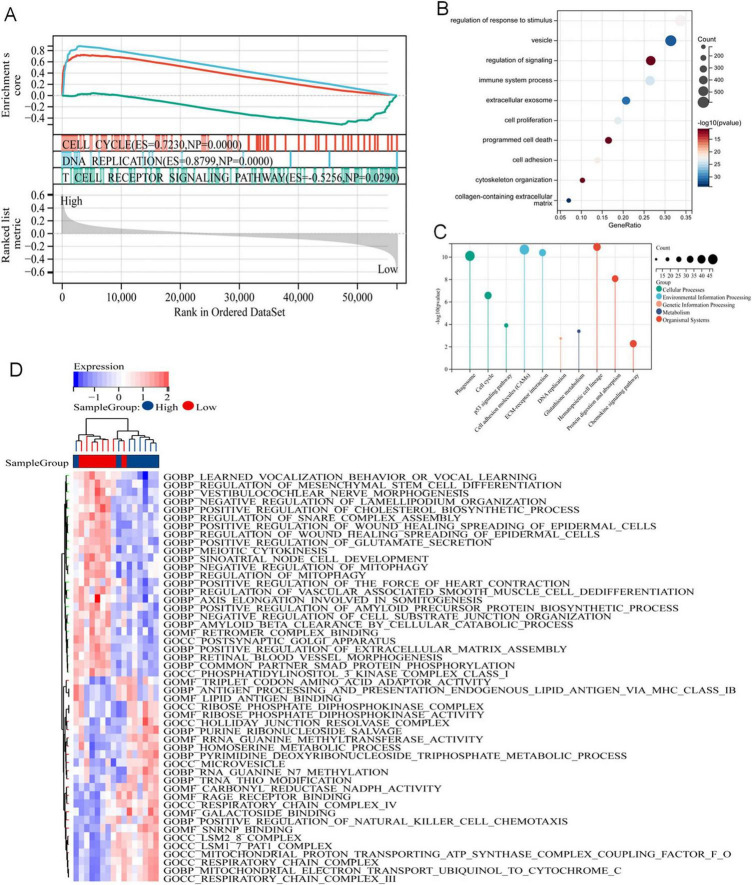
Potential mechanism of DERRDGs in LUAD. **(A)** GSEA enrichment analysis between high-risk subgroups and low-risk subgroups. GO **(B)** and KEGG **(C)** analysis of the differentially expressed genes. **(D)** GSVA enrichment analysis.

To elucidate the pathways associated with these prognostic signals in LUAD, GSEA, GO and KEGG pathways and GSVA analyses were performed. GSEA analysis revealed that these genes were abundant in biological processes related to cell proliferation, including cell cycle and DNA replication, and were related to T-cell receptor signaling pathways (Figure 5A). We screened DERRDGs for GO (Figure 5B) and KEGG (Figure 5C) analysis and found enrichment in various functions and pathways. GO and KEGG were mainly involved immune system process, cell proliferation correlation, programmed cell death, cell adhesion, cytoskeleton organization, signaling pathway. Meanwhile, the GSVA analysis was visualized through the utilization of heat maps (Figure 5D). This suggested our DERRDGs were strongly associated with characteristics of disulfidptosis, primarily affecting cell proliferation and immune regulation in LUAD patients.

### 3.5 Immune pattern and immunotherapy sensitivity based on DERRDGs RiskScore

**FIGURE 6:**
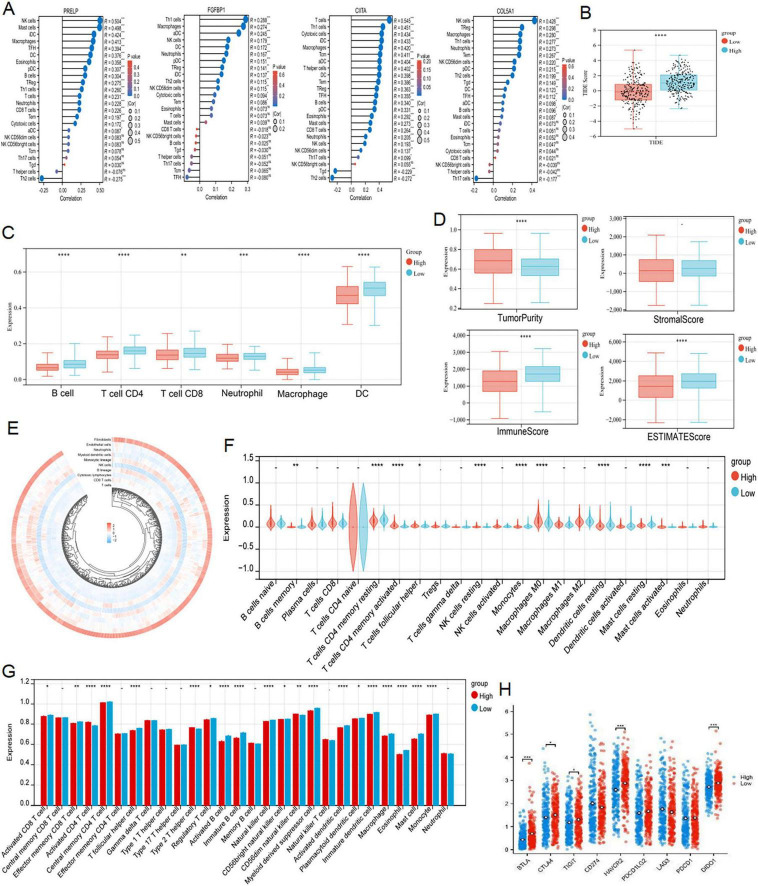
Analysis of immune infiltration characteristics and TIDE score. **(A)** Correlation analysis of the expression levels of 4 DERRDGs in LUAD and the level of immune infiltration. **(B)** Differences in TIDE scores of LUAD patients in different risk groups. TIMER **(C)**, ESTIMATE **(D)**, MCPCounter **(E)** and CIBERSORT **(F)** algorithms between high and low risk subgroups. **(G)** The enrichment of 28 immunoinfiltrating cells in LUAD. **(H)** Comparison of immune checkpoints between high-risk and low-risk subgroups (ns: *P* > 0.05, *P < 0.05, **P < 0.01, ***P < 0.001, ****P < 0.0001).

As GO, KEGG and GSEA results all showed that the genes associated with our prognostic traits were involved in immune infiltration, we persisted in delving into the varying immune microenvironments associated with the DERRDGs RiskScore. At the same time, the four model genes exhibited a profound association with immune cell infiltration, indicating their significant role in the process (Figure 6A). TIDE scores indicated a higher probability of immune evasion in high-risk groups, suggesting a lower probability of patients benefiting from immunotherapy (Figure 6B). The enrichment of 28 immune infiltrating cells in tumor samples was evaluated by ssGSEA. The DERRDGs RiskScore was highly correlated with various CD4 T cells, most CD8 T cell, most helper cells, most B cells, various dendritic cells, natural killer cells, macrophages, eosinophils, mast cells, and monocytes, as assessed for enrichment of 28 immune infiltrating cells in tumor samples (Figure 6G). The TIMER immunoinfiltration analysis revealed a substantial decrease in B cells, macrophages, dendritic cells, neutrophils, CD4 T cells, and CD8 T cells in the high-risk group compared to the low-risk group (Figure 6C). To verify that the analytical algorithm did not impact the function of the two subgroups, we also used ESTIMATE (Figure 6D), MCP-Counter (Figure 6E), and CIBERSOFT (Figure 6F) algorithms for verifying the stability and robustness of the TIMER results. We also discovered notable disparities in the common immune checkpoints between the two subgroups, as depicted in Figure 6H. Taken together, LUAD high-risk patients exhibited higher tumor purity, increased immune cell infiltration, and poorer prognosis.

### 3.6 Somatic mutation

**FIGURE 7:**
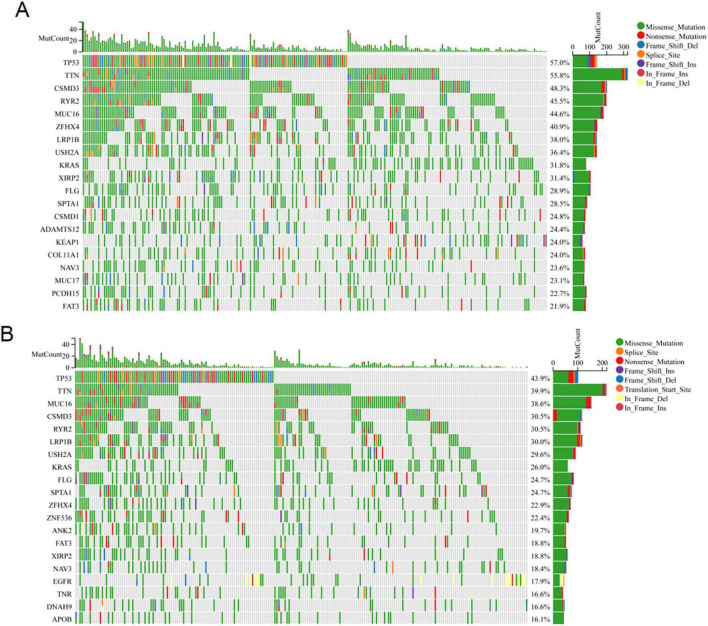
Somatic mutation analysis. The waterfall map shows the genes with the most frequent somatic mutations in high-risk **(A)** and low-risk **(B)** populations.

Extant research has unequivocally demonstrated a profound correlation between the accrual of genetic mutations and the progression of tumorous growths (25, 26). We conducted an analysis of the somatic cell landscape in the TCGA-LUAD cohort, and presented the top 20 genes with highest mutation frequency in the tumor samples through the waterfall diagram. Among individuals in the high-risk category (Figure 7A), the most commonly mutated genes were TP53 (53%), TTN (55.8%), CSMD3 (48.3%), and RYR2 (45.5%). In the low-risk category (Figure 7B), the most commonly mutated genes were TP53 (43.9%), TTN (39.9%), MUC16 (38.6%), and CSMD3 (30.5%).

### 3.7 Clinical applications for nomogram

**FIGURE 8:**
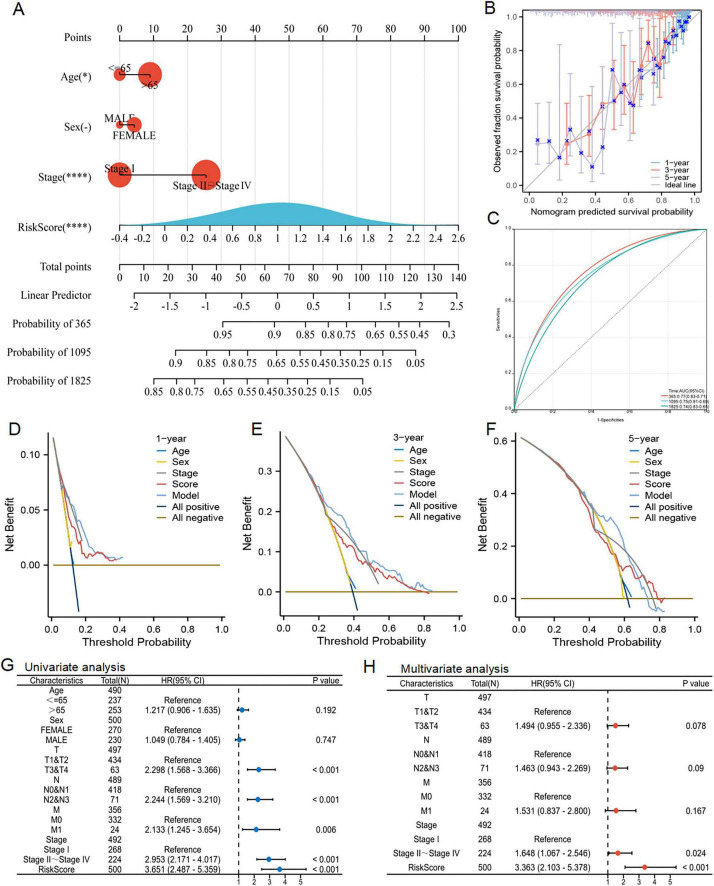
Nomogram prediction model and independent prognosis analysis. **(A)** Construction of a nomogram. **(B)** Evaluation of the calibration curve on the predicted value of the nomogram. **(C)** Evaluation of ROC curve on the predicted value of the nomogram. **(D–F)** Evaluation of the clinical utility value of the DCA curve for the nomogram. **(G,H)** Forest maps showed the results of univariate and multivariate cox regression analysis.

Utilizing the acquired clinical data files and risk profiles, we constructed a nomogram to forecast the survival likelihood of patients at the 1, 3 and 5 year marks (Figure 8A). The results of the calibration curve and ROC curve analysis revealed that the nomogram exhibited a reasonable level of precision in predicting survival, further validating its effectiveness in this regard (Figures 8B, C). The DCA decision curve analysis confirmed that the nomogram had superior efficacy in forecasting the likelihood of survival at the 1 year (Figures 8D–F). Later on, we performed a univariate COX regression analysis, combining RiskScore with clinical features, and the results showed that T, N, M, pathological stage and RiskScore were independent prognostic factors (Figure 8G). Subsequently, based on univariate COX analysis, pathological stage and RiskScore were selected for multivariate COX analysis, revealing them as significant independent prognostic factors (Figure 8H). In summary, the RiskScore was an independent and prognostic indicator.

### 3.8 Identifyng the key DERRDGs gene PRELP

**FIGURE 9:**
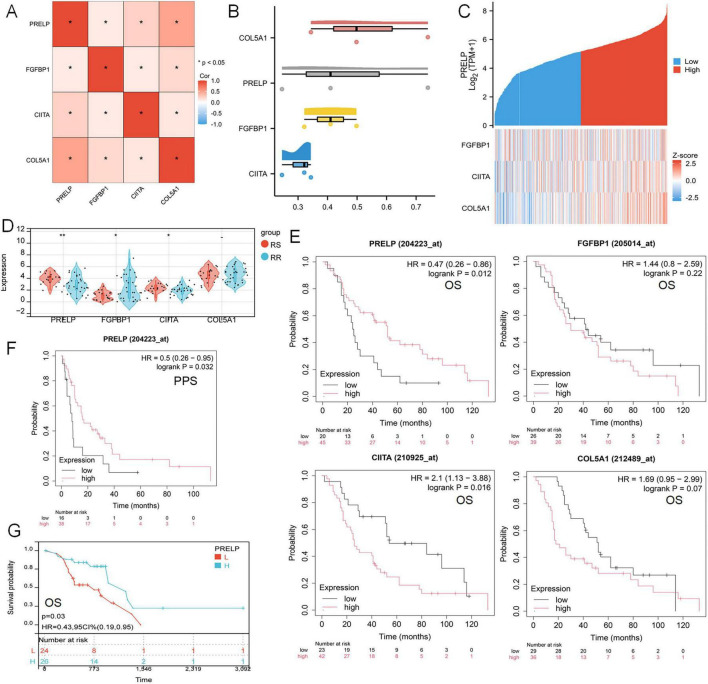
Influence of key gene PRELP on radiotherapy efficacy in LUAD patients. **(A)** Mapping of correlations between PRELP and the other three genes. **(B)** FRIEND analyzed the similarity of the four model genes. **(C)** Heat maps of model gene correlation with PRELP expression. **(D)** Expression of four model genes in TCGA-LUADradiotherapy cohort. **(E)** Survival analysis curve of the relationship between the expression levels of PRELP, FGFBP1, CIITA, COL5A1 and OS in radiotherapy patients reflecting LUAD. **(F)** The survival curve of the relationship between PRELP expression level and PPS was analyzed. **(G)** KM map showed the relationship between OS and PRELP expression levels in TCGA-LUAD radiotherapy patients. (ns: *P* > 0.05, *P < 0.05, **P < 0.01).

The correlation heat map distinctly demonstrated a strong correlation among four model genes: PRELP, FGFBP1, CIITA, and COL5A1 (Figure 9A). Then, FRIEND analysis was performed, in which COL5A1 had the highest similarity (0.52718), followed by PRELP (0.46515) (Figure 9B). The greater the similarity a gene exhibited, the stronger its correlation with other genes, thereby increasing the probability to play a pivotal role. The investigation of PRELP revealed that as the expression of PRELP escalated, the expression of the other three genes similarly underwent varied degrees of change (Figure 9C). Using the TCGA-LUAD-radiotherapy cohort, we discovered that the expression of PRELP and CIITA was significantly higher in RS than RR, while FGFBP1 showed the opposite tendence (Figure 9D). Survival analysis showed radiotherapy patients in the high-expression of PRELP group had a better prognosis, while those in the high-expression group of CIITA had a worse prognosis (Figure 9E). As shown in Figure 9F, this was statistically significant in the post-progression survival (PPS) analysis. Moreover, the same was true of PRELP in the TCGA-LUAD-radiotherapy queue (Figure 9G). These results suggested that we could identify the key gene PRELP associated with radiation-associated disulfidptosis and can speculate that PRELP promotes radiosensitivity in LUAD.

### 3.9 WGCNA analysis and single cell sequencing analysis

**FIGURE 10:**
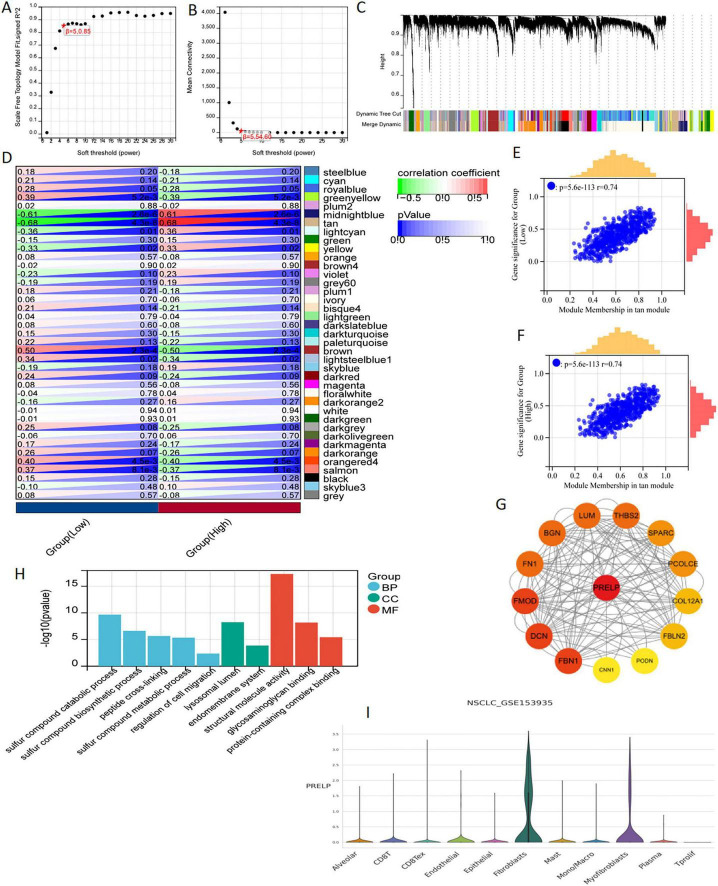
WGCNA analysis and single cell sequencing of PRELP. **(A)** The correlation between the soft threshold and the scale-free topology model is fitted with signed R2. **(B)** Correlation between soft thresholds and average connectivity. **(C)** clustering of module feature vectors. **(D)** The correlation between modules and high or low PRELP expression was calculated. **(E,F)** High correlation between GS and MM in tan modules in high and low expression subgroups. **(G)** PPI showed the correlation genes between tan module Hub gene and PRELP. **(H)** GO enrichment analysis of 14 genes in PPI map. **(I)** In the GSE153935 dataset, the expression of PRELP was different in different cell populations.

Setting β to 3 (*R* = 0.89) in WGCNA offered an appropriate level of power for co-expression network (Figures 10A, B). After identifying six distinct modules, we individually calculated the connection between various color modules and clinical characteristics (Figures 10C, D). Tan module and PRELP high-low expression subgroup exhibited the strongest correlation in terms of the module-trait relationship. The correlation coefficients (*R*-values) for the tan module between Gene Significance (GS) and module membership (MM) were both 0.74 (Figures 10E, F), indicating that the module had a well-organized structure and was significantly associated with prognosis. Hub genes were extracted from tan module. There were 89 genes | MM| > 0.8 and | GS| > 0.1, which were considered to be related to disulfidptosis associated with radiotherapy. At the same time, these Hub genes were analyzed with PRELP on the string platform, and 14 directly related genes were found, as shown in Figure 10G. Moreover, GO analysis for these 14 genes was mainly enriched in the synthesis and decomposition process of sulfide, peptide crosslinking and cell membrane system (Figure 10H).

Using data from the TISCH website, we conducted a thorough analysis of the differential expression patterns of DERRDGs within diverse immune cell subpopulations within the GSE153935 database. Among them, high expression of PRELP was observed in alveolar, CD8 T, endothelial, epithelial, fibroblasts, mast and myofibroblasts, indicating that high expression of PRELP may be closely linked to the immune response in LUAD (Figure 10I). These all suggested that PRELP was closely related to the characteristics of disulfidptosis, primarily affecting immune regulation in LUAD patients.

### 3.10 Validation the expression of DERRDGs in radioresistant cell of LUAD

**FIGURE 11:**
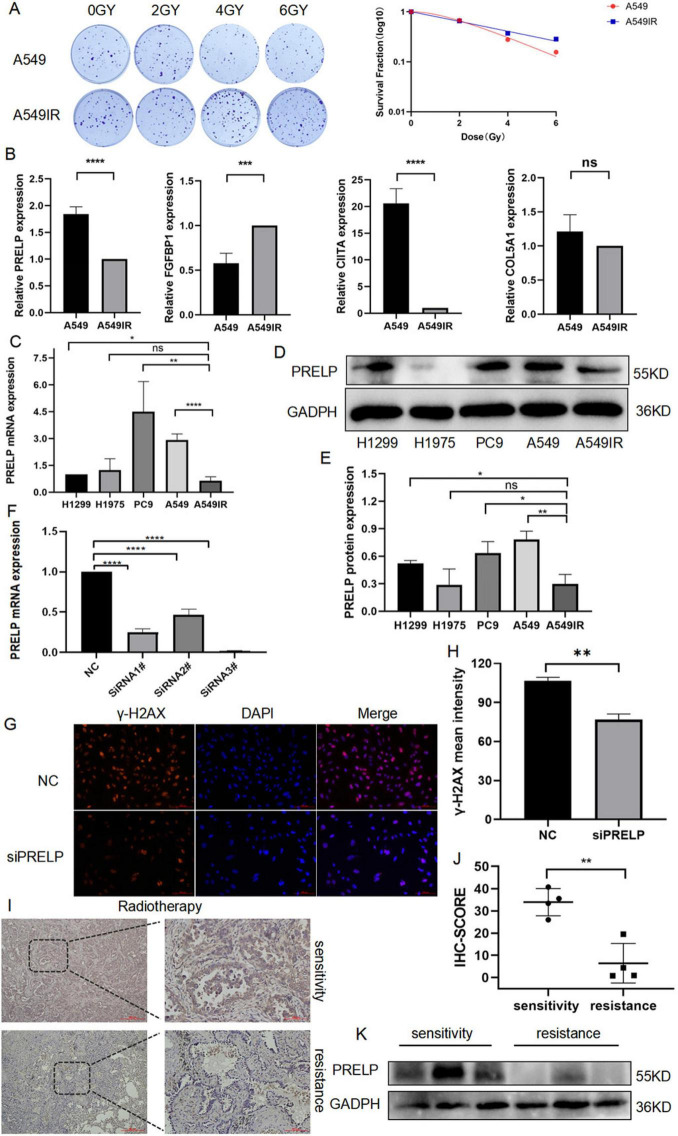
The role of PRELP in the radiotherapy of LUAD. **(A)** Colony formation compared the radiation resistance ability of A549IR with A549. **(B,C)** q-PCR detected the expression of PRELP, FGFBP1, CIITA, COL5A1 in A549 and A549IR as well as in different LUAD cell lines. **(D,E)** WB tested the expression of PRELP in LUAD cell lines. **(F)** Validation the knockdown efficiency of siRNA. Representative images **(G)** and quantitative results **(H)** of γ-H2AX immunofluorescence staining. PRELP expression was obtained by radiosensitive and resistant patients, representative images **(I)** and quantification **(J)** with IHC staining, and WB detection **(K)**. (ns: *P* > 0.05, *P < 0.05, **P < 0.01, ***P < 0.001, ****P < 0.0001).

A549IR was constructed by interval irradiation of A549 parent cells. In this study, we found that the radiation-resistant cell line had a radiation-resistant ability in the clone formation experiment, and the survival rate of its irradiated cells was significantly higher than that of the primary cells (Figure 11A). To further investigate the relationship between prognostic DERRDGs markers and radiotherapy resistance in LUAD, we performed qPCR analysis for A549 and A549IR. The results showed that the expression of FGFBP1 was significantly increased in A549IR, while the opposite trend was observed in PRELP and CIITA (Figure 11B). Subsequently, we further examined PRELP at RNA and protein levels in four different LUAD cell lines and radiotherapy resistant cell line A549IR. Surprisingly, PRELP showed the lowest expression in the resistant cell line at the RNA level (Figure 11C), consistent with the results of our bioinformatic analysis. And WB result also showed the low expression of PRELP in A549IR (Figures 11D, E). In order to further understand the role of PRELP in the progression of radiotherapy resistant in LUAD, we knocked down the expression of PRELP by small interfering, and q-PCR detected that SiRNA3# owed the best effect, which was chose for the following experiment (Figure 11F). γ-H2AX staining experiments clearly showed that down-regulation of PRELP significantly reduced DNA damage after radiotherapy (Figures 11G, H).

We also validated PRELP expression in radiotherapy tumor tissue. We collected pathological specimens of LUAD patients, who were divided into remission and progression groups according to their efficacy evaluation after radiotherapy. We first performed IHC analysis, and the results showed that PRELP was abundant significantly in the remission group compared with the progression group (Figures 11I, J). And our WB analysis also supported above results (Figure 11K). This clearly highlighted the role of PRELP proteins in the progression of resistance to LUAD.

## 4 Discussion

Radiotherapy has been extensively employed in eradicating cancer cells among patients diagnosed with localized LUAD. However, most patients eventually progressed after radiotherapy. Owing to congenital resistance or developed radiation resistance, the invasive growth of tumors is expedited (27, 28). Radiotherapy mainly causes DNA damage, activates cell cycle checkpoints, and leads to cell cycle arrest, thus promoting damage repair (29, 30). Furthermore, radiotherapy boasts the ability to not just diminish tumor size, but also trigger anti-tumor immunity and modify the TME (31, 32). Recently, Liu et al. first proposed a new form of cell death of disulfide called disulfidptosis (9). At present, the specific mechanism of disulfidptosis related genes in LUAD is still not clear, especially in radiotherapy.

In this study, we first analyzed radiotherapy differential genes through GO and KEGG enrichment, and found that radiotherapy was involved in actin cytoskeleton organization, sulfur compound binding and oxidoreductase activity, acting on NAD(P)H, oxygen as acceptor. At the same time, we also performed GO and KEGG enrichment analysis on 24 genes of disulfidptosis in LUAD, which were abundant in regulation of actin cytoskeleton organization, iron-sulfur cluster binding, and focal adhesion. Certainly, we reasonably suspected that radiotherapy was closely associated with disulfidptosis. Next, taken the intersection of DRGs and DEGs and we got 88 DERRDGs. To investigate the correlation between the expression of DERRDGs and the prognosis of LUAD patients, we developed a lasso regression model and single multi-factor model based on the expression profile of DERRDGs in TCGA, and finally obtained 4 DERRDGs, namely PRELP, FGFBP1, CIITA, and COL5A1.

The RiskScore analysis categorized patients in the TCGA-LUAD cohort into two distinct subgroups: a high-risk group and a low-risk group. Kaplan–Meier curve revealed that the OS in high-risk group was significantly lower compared to low-risk group, indicating the effectiveness of this model in predicting the prognosis of LUAD. When examining the time-dependent ROC curve, the area beneath the curve revealed a significant level of precision in the prognosis model’s ability to predict the outcome of LUAD. Consistent results were also obtained in the external GSE50081 and GSE72094 cohorts. To further enhance clinical utilization, a nomogram was crafted and its precision was subsequently validated through rigorous calibration. Then, we assessed the prognostic impact of the model using diverse methodologies, and the results showed that the model had a certain predictive effect on the survival prognosis of LUAD patients in 1, 3, and 5 years.

Interestingly, during the model gene analysis, we found the PRELP gene, which showed a correlation between low expression and a negative prognosis in LUAD. PRELP, a proline/arginine-rich end leucine-rich repeat protein, is a small leucine-rich proteoglycan (SLRP) (33). As a tumor suppressor in solid tumors, PRELP was associated with the occurrence and development of various cancers (34–38). PRELP can inhibit TGF-β and enhance cell-cell adhesion, thereby effectively hindering the progression of cancer (34). TGF–β can affect radiation sensitivity by affecting DNA damage and eliminating cell cycle arrest (39). Our bioinformatics study revealed a decrease in PRELP expression in patients with RR, promoting cell disulfidptosis, and patients with lower PRELP expression had poorer prognosis. Consistently, our real-world results from WB, PCR, and IHC also confirmed that PRELP was down- expressed in resistant cells or tissues. The results of our GSEA, GO, and KEGG analyses were significantly correlated with the cell cycle. We can boldly speculate that PRELP affected the treatment efficacy by increasing the tolerance to radiotherapy through disulfidptosis and cell cycle. Nevertheless, the biological role of PRELP in radiotherapy of LUAD has not been elucidated, and the precise mechanism by which it triggers disulfidptosis requires additional experimental investigation.

Radiotherapy can not only cause tumor damage, but also change the proportion of immune cell infiltration by reshaping the immune microenvironment of the tumor (40, 41). In our study, the results of GSEA, GO and KEGG analysis exhibited significantly correlated with immune response-related pathways, mainly including immune system processes, T-cell receptor signaling pathways and so on. Therefore, we speculated that disulfidptosis activity could alter the microenvironment infiltration of immune cells by modulating various immune systems, which could be used as a novel immunomodulatory strategy to improve the efficacy of radiotherapy in LUAD patients. Our study analyzed the infiltration of immunological microenvironments in various risk groups and identified notable disparities in B cell, CD4 T cell, CD8 T cell, neutrophil, macrophage and DC. It was postulated that TIDE scores served as indicators of the likelihood of tumor immune evasion, thereby implying that elevated TIDE scores in high-risk group could account for the unfavorable prognosis observed within this demographic (42). Our study additionally discovered that PRELP expression was positively correlated with the number of CD8 T cells, which play an anti-tumorigenic development and immune elimination role in LUAD. Recent studies have found that PRELP could affect tumor immunogenicity in melanoma by up-regulating MHC Class I surface expression, thereby inhibiting tumor development and enhancing CD8 T cell infiltration (34), which was consistent with our findings. Cytotoxic T lymphocytes (CTL cells), often referred to as CD8+T cells, are a key component of the adaptive immune system and play an important role in the immune system’s defense against pathogens such as viruses, bacteria, and tumors (43). There is increasing evidence that radiation therapy can induce a tumor-specific CD8+T cell response, which is essential for radiation-mediated tumor regression (44, 45). So, we hypothesized that PRELP regulated immune regulation of CD8+T cell infiltration after radiotherapy. Although there is a limited amount of research on the correlation between disulfidptosis and tumor immunity, further investigation is required to understand its mechanism.

This study should acknowledge a number of limitations. The model mainly relied on TCGA databases and GEO databases, so its generalization to other datasets may be limited. And more experiments should be employed to elucidate the mechanism.

## 5 Conclusion

This study has found, for the first time, four genes related to disulfidptosis that are associated with the radiation and prognosis of LUAD, and explored their potential molecular mechanisms, establishing an effective prediction model for LUAD prognosis. Finally, we also identified the key gene PRELP, which has potential significance for improving the efficacy and prognosis of radiotherapy in LUAD patients. To summarize, our research results may offer novel insights into the different subtypes of LUAD and their relationship with radiation.

## Data Availability

The original contributions presented in this study are included in this article/supplementary material, further inquiries can be directed to the corresponding authors. Publicly available datasets were analyzed in this study. This data can be found here: section “2.1 Data collection and sample processing.”

## Footnotes

1
https://www.genome.ucsc.edu/

2
https://www.ncbi.nlm.nih.gov/geo/

3
https://cn.string-db.org

4
http://cis.hku.hk/TISIDB/

5
https://kmplot.com/analysis/

6
http://tisch1.comp-genomics.org/
